# Notes and Notices

**Published:** 1898-07

**Authors:** 


### NOTES AND NOTICES.

DR. J. S. BELL, of Rock Valley, Iowa, has removed to 2200 Congress Street, Chicago, Ill.

FOR SALE.—Vols. 12, 13 and 14 of THE HOMŒOPATHIC PHYSICIAN, unbound.

WM. STEINRAUF, M. D., St. Charles, Mo.

THE MONUMENT TO HAHNEMANN, unequaled in this country and unsurpassed anywhere as a work of art—already famous—is now nearly completed. The granite work from the quarries of the Maine and New Hampshire Granite Co. has been finished with the exception of some of the finer carving and the lettering. The statue and bas reliefs have been cast in bronze by the Gorham Mfg. Co., and will be exhibited at the several art exhibitions this season in New York. It is not designed alone to honor the leader of a great reformation and founder of a school of medicine, but also as an enduring monument to the stability and growth of our method of cure, directing general attention to our existence, exerting an influence on local recognition and legislation, and strengthening our position everywhere in this and other lands.

The treasurer of the fund, Henry M. Smith, M. D., 288 St. Nicholas Avenue, New York, is out in a circular urging every member of the profession to send him a uniform subscription of five dollars ($5.00), to enable him to complete the amount necessary to pay for the monument.

THE HOTEL LITHIA, Tallapoosa, Georgia, is a modern and thoroughly substantial Hotel, finished throughout with hard-wood, and supplied with the latest improved plumbing. Rooms are lighted with electricity, heated with steam, and many of them with open grates. The rooms are single or in suites of two or more, a number of the suites having private bath and dressing rooms attached. Electric bell to each room. The furniture is elegant, beds supplied with hair mattresses and improved springs.

Elevator, baths, billiard rooms, and ball room; spacious halls and rotundas on every floor; over 400 feet of 14-foot verandas; large balconies on each floor, and an observatory commanding a view of the surrunding country for fifty miles. The dining-room, "with truss girders and no posts," is ample to seat 250 guests. The hotel will be conducted in a strictly first-class manner, on the American plan, at reasonable rates. Invalids traveling for their health will find in this hotel all the comforts of a home. Pure spring water used for drinking and cooking. A resort for health and pleasure, winter and summer, in the mountains of Northwest Georgia, on the southern extremity of the Blue Ridge range and Piedmont plateau, on the Southern Railway, in the heart of a longleaf pine and hard-wood district, 1,200 feet above the sea. The climate, winter and summer, cannot be surpassed by any section of the United States. While not as warm and enervating as the sub-tropical climate of Florida, in the winter it is mild, with continuous days of sunshine and scarcely ever a fall of snow. It is a happy medium between the warm, heavy, enervating climate of Florida and the severe cold of the North, giving at once to the nervous and sleepless and to the over-worked brain and body a soothing and restful influence. It seems to be Nature's own sanitarium. Northern physicians send their patients here for recuperation. Throughout the summer the nights are cool, and it is an exception when blankets are not needed for comfort. Mean temperature, spring and fall, 68 degrees: summer 71 degrees; January (the coldest month) 43 degrees. Annual temperature, as determined from Government observations, 61.5 degrees. No unhealthy marshes or swamps, and the best of freestone water abounds in inexhaustible springs in hillsides and valleys. The absolute healthfulness of the locality is attributable to the high elevation, pure water, perfect drainage, pine-laden air, absence of swamps and limestone, no excess of heat or cold or extreme changes of temperature, and no silurian or limestone valleys.—*E. H. Holbrook, M. D., Manager.*

A Great Reward: To the Editor of *The Times*:

The accompanying little story was sent me by a niece in the West, and was written by a little boy in Denver, one of a class of children of six or eight years old, who had been requested by their teacher to write a story, they to select a subject, and their compositions not to be subject to revision by their teacher, but to be read before the children's parents exactly as written. This is one of the number submitted, and I thought it might amuse your readers. Yours truly, C. J. Price.

A poor young man fell in love with the daughter of a rich lady, who kept a candy shop. The poor young man could not marry the rich candy lady's daughter because he had not enough money to buy any furniture.

A wicked man offered to give the young man twenty-five dollars if he would become a drunkard. The young man wanted the money very much, so he could marry the rich candy lady's daughter, but when he got to the saloon he turned to the wicked man and said: "I will not become a drunkard, even for great riches. Get thee behind me, Satan."

On his way home he found a pocket-book containing a million dollars in gold; then the young lady consented to marry him. They had a beautiful wedding, and the next day they had twins. Thus you see that "virtue has its own reward."—Philadelphia *Times*, April 13th, 1898.

